# Quantifying Canopy Closure Dynamics Using UAV Imagery and Semantic Segmentation in Rice Breeding Trials

**DOI:** 10.3390/plants15121860

**Published:** 2026-06-16

**Authors:** Yue Bao, Fudeng Huang, Weidong Lou, Ying Zhu, Xiaobin Zhang, Qing Gu

**Affiliations:** 1 College of Mathematics and Computer Science, Zhejiang A&F University, Hangzhou 311300, China; baoyue6638@163.com; 2 Institute of Digital Agriculture, Zhejiang Academy of Agricultural Sciences, Hangzhou 310021, China; louwd@zaas.ac.cn; 3 Institute of Crop and Nuclear Technology Utilization, Zhejiang Academy of Agricultural Sciences, Hangzhou 310021, China; pahfd@126.com; 4 Institute of Virology and Biotechnology, Zhejiang Academy of Agricultural Science, Hangzhou 310021, China; yzhuzaas@163.com

**Keywords:** rice (*Oryza sativa* L.), canopy closure dynamics, UAV remote sensing, deep learning, semantic segmentation, Gompertz model

## Abstract

The canopy closure stage is a critical phase of rice (*Oryza sativa* L.) development that influences canopy structure and final grain yield. Accurate and continuous monitoring of canopy closure dynamics is therefore essential for variety screening and cultivation optimization. This study combines unmanned aerial vehicle (UAV) remote sensing technology with deep learning-based semantic segmentation to establish an efficient framework for quantifying rice canopy closure dynamics. UAV RGB images were acquired for 198 hybrid rice varieties during early growth stages and used to build a canopy segmentation dataset. Three semantic segmentation models, i.e., DeepLabv3+, U-Net, and PSPNet, were systematically evaluated. Results show that DeepLabv3+ performed the best and enabled precise extraction of rice canopy features, obtaining a mean intersection over union (mIoU) of 0.86. Based on the extracted canopy coverage, the Gompertz model was utilized to characterize temporal canopy closure trajectories for all varieties, achieving an average R^2^ of 0.978. Subsequently, five key dynamic indicators were derived, including canopy closure limit value (*K*), initial growth coefficient (*a*), growth rate coefficient (*b*), maximum instantaneous growth rate (*MGR*), and days to maximum growth rate (*Tm*). K-means clustering analysis was performed on these indicators to categorize all rice varieties into three clusters, disclosing pronounced differences in early-stage canopy development characteristics. Correlation analysis further demonstrated that canopy closure dynamics were closely associated with grain yield. Overall, while acknowledging the limitations of a single-season and single-site dataset, this study provides a scalable and objective framework for quantifying rice canopy closure dynamics, offering valuable support for variety selection, cultivation optimization, and high-yield rice production.

## 1. Introduction

Rice (*Oryza sativa* L.) is one of the three major staple food crops worldwide and serves as the primary food source for more than half of China’s population. Ensuring stable rice production is therefore directly related to China’s national food security and the sustainable development of agriculture [1]. During rice cultivation, the canopy closure period represents a key growth stage that reflects population growth status and plays a crucial role in determining final yield performance [2]. Consequently, the accurate characterization of canopy closure dynamics across different rice varieties is of both theoretical significance and practical importance for superior variety screening, cultivation management optimization, and the development of high-yield and high-efficiency planting models.

The canopy closure stage marks a critical transition from vegetative growth toward reproductive development in rice. This stage is characterized by pronounced temporal dynamics that not only reflect varietal genetic traits but are also strongly influenced by agronomic practices and environmental conditions, including water and fertilizer management, planting density, and local ecological factors [2]. Appropriate canopy closure dynamics enable rice populations to rapidly establish a favorable canopy structure during the tillering stage, ensuring sufficient photosynthetic capacity for biomass accumulation. At the same time, they help to avoid adverse effects associated with premature or excessive canopy closure, such as poor ventilation, reduced light penetration, and increased risks of pest and disease occurrence.

Rice canopy closure dynamics are traditionally monitored based on manual field surveys, where indicators like canopy coverage, plant height, and tiller number are visually estimated or measured to evaluate the canopy closure process. However, this method has obvious limitations: it is inefficient and difficult to implement for large-scale, multi-period monitoring. Moreover, it suffers from large subjective errors and cannot capture three-dimensional (3D) structural information within the canopy, which makes it difficult to precisely quantify subtle changes. As modern agriculture transforms toward precision and intelligence, traditional methods can no longer meet the requirements of large-scale variety screening and field management optimization, which makes it necessary to develop efficient, precise, and non-destructive monitoring technologies [3].

The rapid development of unmanned aerial vehicle (UAV) remote sensing technology has provided a novel solution for crop phenotypic monitoring. The outstanding advantages of UAVs in terms of flexibility and portability allow them to collect high-resolution imagery across high-frequency temporal dimensions and large-scale spatial dimensions. Consequently, they serve as an ideal tool for achieving high-throughput and non-destructive crop phenotypic monitoring [4,5]. As a UAV is equipped with multispectral and RGB cameras, it can quickly acquire rice canopy imagery and provide data support for wide-range, high-frequency monitoring. Simultaneously, the rapid development of deep learning technology provides a powerful precise image analysis technique. Its excellent image segmentation, feature extraction, and object recognition capabilities allow it to automatically extract feature parameters relating to canopy closure status from complex canopy images, which can enable a quantitative analysis of the canopy closure dynamics [6].

In recent years, deep learning has gained widespread adoption in agricultural remote sensing, yielding significant advancements, particularly in vegetation coverage segmentation. This success is primarily attributed to its robust capacity for modeling complex nonlinear relationships and extracting high-level abstract features [7]. For instance, Jin et al. [8] employed image classification neural networks to detect and estimate weed coverage in turf. Tanabe et al. [9] acquired UAV multispectral imagery across four wheat growth stages to extract spectral data and coverage-related information, such as plant morphology and canopy structure; these features were subsequently utilized to develop a convolutional neural network (CNN) model for yield prediction. Similarly, Bai et al. [10] leveraged deep learning models to capture the nonlinear relationships between variables such as canopy coverage and leaf age, achieving rapid, non-destructive, and high-precision estimation of maize leaf age. Furthermore, Sato et al. [11] employed multiple linear regression to construct a coverage estimation model for large-scale rice monitoring. Collectively, these studies validate the feasibility and superiority of integrating UAV remote sensing with deep learning for crop phenotypic monitoring.

Dynamic analysis is a quantitative research method based on time-series data. It refers to the process of discovering patterns, trends, and critical transition points of a subject’s evolution over time through the continuous collection of status data at various time nodes. Xie et al. [12] used five machine learning methods to extract rapeseed flowering areas and define relevant dynamic phenotypic parameters. The authors used K-means clustering to classify all rice varieties into different clusters, comparing significant differences in multiple phenotypic parameters across different clusters to achieve rapid, objective and large-scale monitoring of rapeseed flowering dynamics. Zhang et al. [13] used data from key wheat growth stages to extract features and perform spatiotemporal modeling, achieving precise monitoring of the entire wheat growth cycle.

Although previous studies have demonstrated the utility of UAVs and deep learning in crop phenotyping, most have prioritized static coverage estimation at discrete growth stages, rather than capturing the continuous, high-frequency progression of canopy closure. This study integrates multi-temporal RGB imagery to quantify rice canopy closure and employs the Gompertz model to derive dynamic indicators that differentiate hybrid rice varieties based on their early-growth patterns. The core hypothesis is that UAV-based continuous monitoring can effectively capture distinct canopy closure dynamics among varieties, and that these early dynamic traits are significantly associated with final grain yield. To test this, we addressed the following research questions: (1) Which semantic segmentation model performs best for high-frequency, complex rice canopy extraction? (2) Can Gompertz modeling and K-means clustering objectively categorize rice varieties based on their early canopy development? (3) How do these distinct dynamic traits correlate with final agronomic yield? Ultimately, this study establishes an efficient framework for monitoring rice canopy closure dynamics, providing actionable insights for variety selection, cultivation optimization, and high-yield rice production.

## 2. Results

### 2.1. Model Training and Performance Comparison

#### 2.1.1. Performance Comparison of Different Models

The performance of the three semantic segmentation models was evaluated using key metrics calculated on the validation set. As shown in Table 1, the DeepLabv3+ model exhibited superior performance, achieving a mean intersection over union (mIoU) of 0.860. This represents a relative mIoU improvement of 1.77% and 32.92% over U-Net (0.845) and PSPNet (0.647), respectively. Furthermore, DeepLabv3+ achieved a mean pixel accuracy (mPA), mPrecision, mRecall, and Macro-F1 score of 0.925, 0.922, 0.925, and 0.924, respectively, underscoring the balanced and robust segmentation capabilities of the DeepLabv3+ architecture. The U-Net model followed closely with an mIoU of 0.845, and all other metrics remained above 0.91, demonstrating a performance nearly comparable to DeepLabv3+. In contrast, PSPNet exhibited significantly lower performance, with all indicators considerably below those of the other two models.

**Figure 1:**
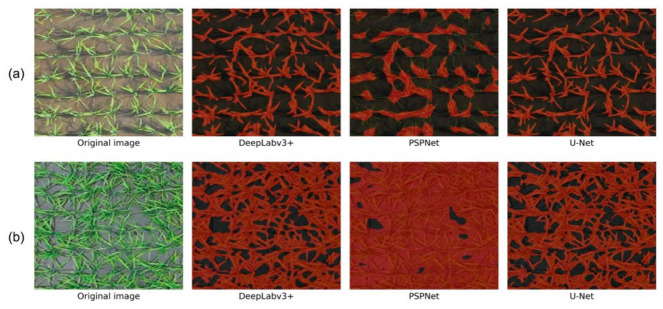
Comparison of rice canopy segmentation results among DeepLabv3+, PSPNet, and U-Net. (**a**) Sparse canopy scenarios at 15 days after transplantation (DAT) (seedling stage, Plot No. 79). (**b**) Dense canopy scenarios at 23 DAT (tillering stage, Plot No. 73). Red masks represent the segmented rice canopy.

Figure 1 presents the visualized segmentation results for typical plots. Figure 1a illustrates a plot during the seedling stage at 15 days after transplantation (DAT). It is evident that DeepLabv3+ accurately identified sparse leaves with negligible omissions. In contrast, U-Net exhibited minor information loss at the leaf edges, while PSPNet erroneously misclassified portions of the soil background as canopy. At the tillering stage (23 DAT), as shown in Figure 1b, both DeepLabv3+ and U-Net successfully captured the continuous canopy areas. However, DeepLabv3+ demonstrated a superior ability to delineate internal gaps within the canopy, whereas PSPNet continued to exhibit noticeable segmentation blurring and boundary misalignment. A synthesis of the quantitative metrics and visual inspections confirmed that the DeepLabv3+ model delivered the optimal overall performance for rice canopy semantic segmentation, thereby providing a reliable data foundation for the subsequent extraction of canopy closure dynamic indicators.

#### 2.1.2. Canopy Segmentation Performance Across Growth Stages (GS)

The segmentation stability and applicability of the optimal DeepLabv3+ model were further verified across nine growth stages (GS1–GS9) using multi-temporal imagery. As shown in Table 2, the DeepLabv3+ model maintained high segmentation accuracy throughout the growth cycle, despite notable stage-specific variations. During the seedling stage (GS1–GS2), canopy coverage was relatively low (30–45%), characterized by sparse, dispersed leaves and a high proportion of background pixels. During this period, the model was influenced by water-surface reflections and canopy shadows, yielding its lowest performance with an mIoU of 0.78–0.81 and an mRecall of 0.85–0.88.

As the rice entered the tillering stage (GS3–GS8), rapid tillering increased the canopy coverage to 60–90%, forming a progressively continuous structure. Consequently, the segmentation performance improved significantly: mIoU stabilized between 0.86 and 0.89, while mPA, mPrecision, and mRecall all exceeded 0.93. Optimal performance was achieved at GS6 (28 DAT, ~85% coverage), with an mIoU of 0.89 and a Macro-F1 of 0.95. At this stage, clear boundaries between the canopy and background, combined with high spatial continuity, allowed the multi-scale feature extraction capabilities of atrous convolution to be fully leveraged, effectively balancing precision and recall while minimizing misclassifications.

By the jointing stage (GS9, 40 DAT), canopy coverage exceeded 95%, leading to severe leaf overlapping and canopy closure. The mIoU decreased slightly to 0.85 and mRecall dropped to 0.91, while mPrecision remained at 0.92. This shift is primarily attributed to spectral information confusion in overlapping areas under high-density conditions, where leaf shadows or overlaps may slightly increase false positives. Nevertheless, the model maintained high overall accuracy, meeting the requirements for canopy closure dynamic analysis.

Overall, DeepLabv3+ exhibited robust adaptability across critical rice growth stages. Notably, the model reliably quantified canopy coverage even under sparse conditions during the early seedling stage, ensuring consistent and stable data support for continuous tracking of canopy closure dynamics throughout the growth cycle.

### 2.2. Canopy Closure Dynamic Analysis

**Figure 2:**
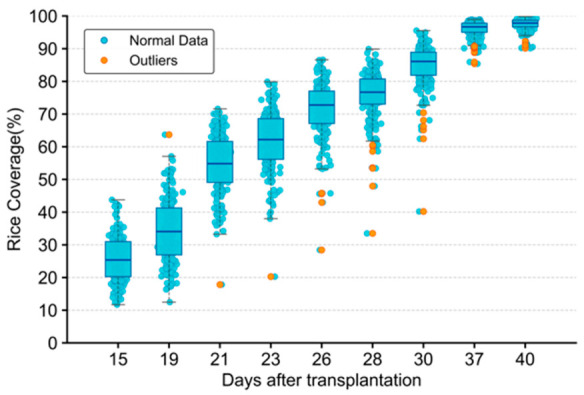
Trend in the dynamic changes of rice canopy closure from 15 to 40 days after transplantation (DAT) across the 198 hybrid rice varieties.

The time-series variation of rice canopy coverage is the core indicator that reflects the canopy closure dynamics. Table 3 shows the fitting results. Figure 2 shows the canopy closure dynamic trends for the 198 rice varieties. The values of R^2^ for all varieties exceeded 0.95, ranging between 0.952 and 0.994 with an average R^2^ of 0.978. This demonstrates that the fitted curves effectively captured the temporal variation patterns of canopy coverage. The root mean square error (RMSE) varied between 1.21% and 3.87% with an average of only 2.36%, and the mean absolute error (MAE) varied between 0.95% and 3.02% with an average of 1.89%, indicating minimal deviation between the fitted and measured values and high predictive accuracy. The seedling and jointing stages primarily exhibited slight variations in fitting performance. In the seedling stage, the RMSE for some varieties was slightly higher and varied between 3.0% and 3.8% due to low canopy coverage and sparse leaves. In the jointing stage, the MAE for a few varieties reached 2.5–3.0% due to canopy closure and severe leaf overlapping.

**Figure 3:**
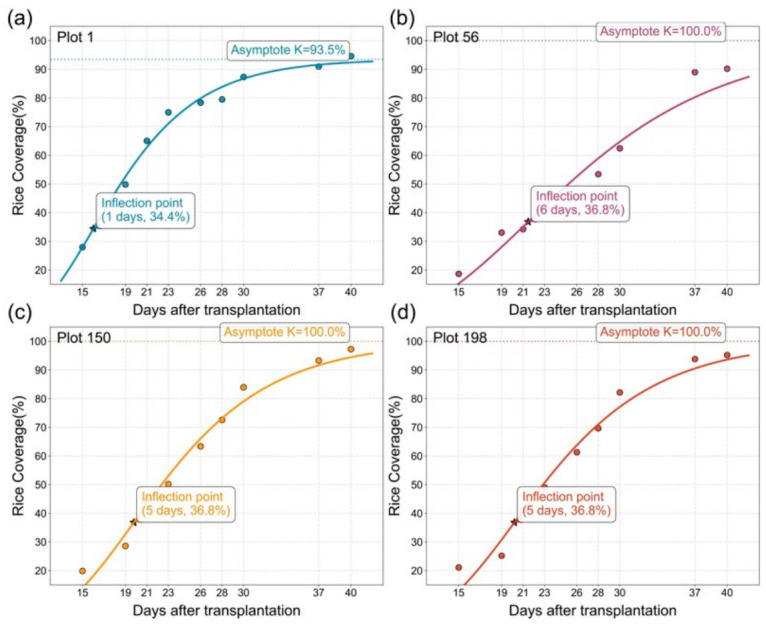
Curve fitting results for four representative rice plots using the Gompertz model. (**a**) Plot 1 (Cluster A); (**b**) Plot 56 (Cluster B); (**c**) Plot 150 (Cluster C); (**d**) Plot 198 (Cluster A). Scatter points represent measured canopy coverage data, and solid lines indicate the fitted growth curves.

Figure 3 presents the superior fitting performance of the Gompertz model for four randomly selected representative varieties in Plots 1, 56, 150, and 198. Specifically, Plot 1 (Cluster A) exhibited rapid early growth, characterized by a steep slope during the tillering stage. Plot 56 (Cluster B) showed a more gradual initial growth rate that intensified during the later stages. Plot 150 (Cluster C) featured an accelerating growth rate, with the curve’s slope peaking during the mid-tillering stage. Plot 198 (Cluster A) displayed a compact overall growth rhythm, where the fitted curve was almost identical to the measured values. This performance was confirmed by an R^2^ of 0.992 and an RMSE of 1.35%. These results fully validate the applicability and reliability of the Gompertz model for fitting rice canopy closure dynamics.

### 2.3. Clustering Analysis of Rice Varieties

**Figure 4:**
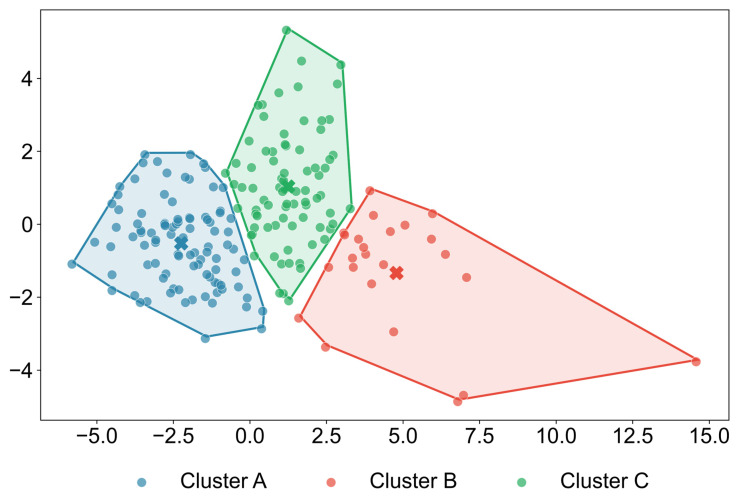
Clustering analysis results of 198 rice varieties based on canopy closure dynamic parameters. The polygons illustrate the spatial distribution and degree of separation among the clusters.

**Figure 5:**
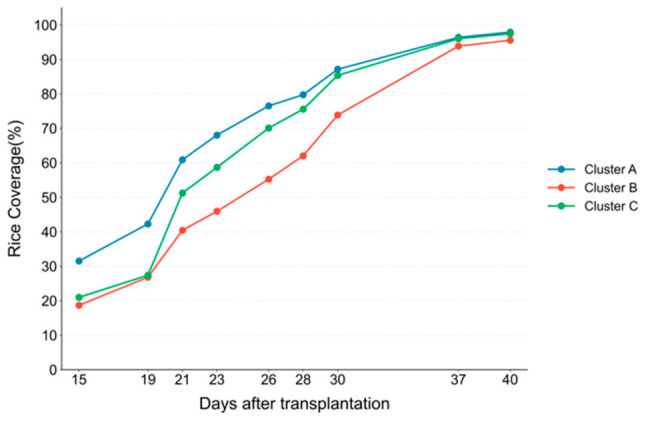
Temporal evolution of rice canopy coverage across the three identified clusters. The curves represent the mean dynamic trends for Clusters A, B, and C, illustrating distinct growth rhythms throughout the observation period.

To investigate the similarity of the rice canopy closure dynamics, K-means clustering analysis was performed based on five dynamic indicators (*K*, *a*, *b*, *MGR*, *Tm*). The optimal number of clusters was determined via the elbow method and further validated by a maximum silhouette score of 0.65, confirming that k = 3 provided the most cohesive and well-separated grouping for the canopy dynamic parameters. As illustrated in Figure 4, the 198 rice varieties were classified into three distinct clusters: A (95 varieties), B (24), and C (79), represented by polygons of different colors. The lack of significant overlap between these polygons indicates clear spatial distinctions among the three clusters. Figure 5 displays the temporal dynamics of canopy coverage for these clusters across different growth stages. Cluster A exhibited the fastest initial growth rate, whereas Cluster C showed a more moderate early growth speed that subsequently converged with Cluster A in the later stages. In contrast, Cluster B exhibited the slowest growth during the early phases but experienced significant acceleration later.

These results suggest that the primary phenotypic divergence among rice varieties lies in their early-stage growth rhythms rather than their final coverage outcomes. From an agronomic perspective, identifying these distinct growth patterns is highly practically relevant. Evaluating early canopy closure efficiency is more discriminative for variety screening than relying on static final coverage. For instance, the strong early vigor of Cluster A enables rapid weed suppression by swiftly shading the soil surface and maximizing radiation interception during the critical tillering stage. Conversely, the slower early growth of Cluster B suggests a potential need for targeted interventions, such as increasing the initial planting densities or applying mid-tillering nitrogen topdressing to compensate for delayed canopy expansion.

**Figure 6:**
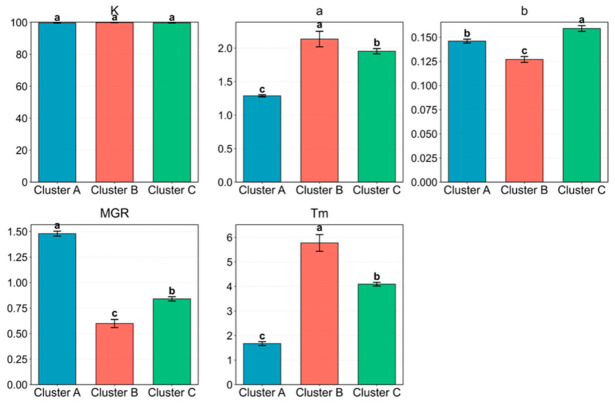
Comparative analysis of canopy closure dynamic indicators among the three identified clusters. Different letters above the bars indicate significant differences among clusters based on Tukey’s HSD test (*p* < 0.05).

Figure 6 shows the comparative analysis of canopy closure dynamic indicators among the three identified clusters. One-way analysis of variance (ANOVA) followed by Tukey’s HSD test revealed highly significant differences (*p* < 0.05) across clusters for parameters *a*, *b*, *MGR*, and *Tm*. Conversely, the *K* value exhibited no significant difference, consistently remaining above 99% for all varieties. This suggests that the difference between clusters is not reflected in the canopy closure endpoint but rather in the growth rhythm. Specifically, the *a* value of Cluster B was significantly higher than those of Clusters A and C, indicating a higher threshold for early growth initiation. Cluster C displayed a significantly higher *b* value, reflecting a more rapid acceleration in growth rate. The *MGR* of Cluster A was significantly greater than those of Clusters B and C, demonstrating superior canopy closure efficiency. Furthermore, the *Tm* of Cluster B was significantly longer, indicating that its growth peak occurred the latest among all groups.

Overall, Cluster A was characterized by the highest *MGR* and the earliest *Tm*, resulting in rapid early-stage canopy development. Cluster B featured the highest *a* and the longest *Tm*, exhibiting a gradual early growth rhythm that prevents excessive vegetative overgrowth. Cluster C displayed the highest *b* value, with other growth indicators intermediate between Clusters A and B, reflecting a superior capacity for steady acceleration and high population uniformity during the mid-tillering phase.

## 3. Discussion

### 3.1. Methodological Prospects

Plant phenotypes directly reflect the interaction between plant genotypes and the environment, encompassing morphological, physiological, and phenological characteristics such as plant height, canopy structure, and growth stage dynamics [14,15]. They provide the core foundation for crop genetic breeding, cultivation management, and yield and quality regulation. Precise phenotypic data facilitate the screening of elite genotypes that exhibit high yield and stress resistance, thereby shortening the breeding cycle. In cultivation management, phenotypic monitoring enables the real-time tracking of crop population growth status, providing a scientific basis for field measures such as irrigation, fertilization, and density optimization, which are of great significance for ensuring food security. However, plant phenotypes are traditionally acquired using manual surveys, which are time-consuming, labor-intensive, and inefficient, making it difficult to meet the demands of large-scale and multi-period dynamic monitoring. In addition, these surveys are also prone to significant errors due to subjective judgments of different surveyors. Consequently, these methods can no longer adapt to the precision and high-throughput needed in modern agriculture research [16]. In contrast, UAV remote sensing technology provides flexibility, convenience, and high resolution. Furthermore, it can carry RGB, multispectral, and other sensors to rapidly acquire large-scale canopy image data. These features allow it to achieve high-frequency and non-destructive dynamic monitoring covering all key growth stages from seedling to maturity, and provide rich data sources for the subsequent extraction of phenotypic parameters. Therefore, UAV technology effectively addresses the limitations of traditional methods, providing key technical support for high-throughput and precise analysis. This paves the way for intelligent and large-scale applications in the field of crop phenotyping [17,18,19].

Traditional manual surveys exhibit significant deficiencies in the acquisition of rice canopy closure data. These methods, which rely on the visual estimation of canopy coverage and manual measurement of plant height, are impractical for high-frequency monitoring across multiple growth stages in large-scale experiments, such as the 198 plots considered in this study. Furthermore, manual methods are prone to substantial subjective errors. As UAV-based imagery has been widely utilized to detect crop growth and estimate traits [20,21,22,23], we utilized a UAV equipped with RGB sensors to collect image data during key growth stages. Imagery of the 198 plots was collected throughout the early growing season at a higher frequency than traditional ground-based surveys. This approach enables rapid and efficient data collection, minimizing the reliance on time-consuming manual field surveys.

**Figure 7:**
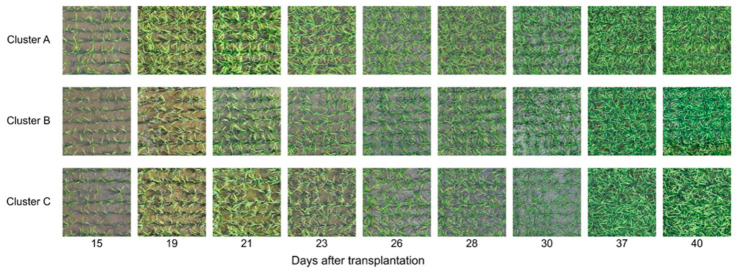
Multi-temporal RGB image patches illustrating the canopy closure progression of representative rice plots from the three clusters.

The rice canopy closure dynamic curves obtained from UAV-based time-series imagery can comprehensively reflect the dynamic coverage changes that occurred throughout the study period [24]. Five indicators, including the canopy closure limit value, initial growth coefficient, and growth rate coefficient, were extracted to characterize these changes. Subsequently, these indicators were used as input variables for the K-means clustering algorithm to classify all rice varieties into three groups. This UAV-based approach enabled the investigation of the growth dynamics of various rice varieties. In this study, the established evaluation index system for canopy closure dynamics serves as an important basis for predicting the high-yield potential of varieties. Its integration with the high-throughput monitoring advantages of UAV remote sensing technology facilitates the precise matching of variety screening and cultivation management, thereby providing technical support for constructing high-yield and high-efficiency planting models. Figure 7 depicts multi-temporal RGB image patches illustrating the canopy closure progression of representative rice plots from the three clusters.

### 3.2. Performance Comparison of Semantic Segmentation Models

In research involving precise rice canopy coverage extraction, the choice of semantic segmentation architecture directly determines the reliability of time-series data. This, in turn, affects the accuracy of canopy closure dynamic indicators and the scientific validity of yield correlation analysis. Significant dynamic variations exist in rice canopy imagery, transitioning from sparsely distributed leaf fragments during the seedling stage to a progressively densifying population structure during tillering, and finally to a closed, overlapping canopy morphology at the jointing stage. These variations in canopy density, spatial distribution, and background interference levels across growth stages impose stringent requirements on a model’s feature extraction capability, anti-interference robustness, and adaptability [25,26,27].

Compared to U-Net and PSPNet, DeepLabv3+ performed superiorly due to its multi-scale sampling via atrous convolution and its encoder–decoder structure. This architecture effectively adapts to dynamic canopy changes, precisely preserving detailed features while minimizing segmentation blur. In this study, DeepLabv3+ demonstrated optimal performance across all metrics, achieving an mIoU of 0.860, with mPA, mPrecision, mRecall, and Macro-F1 score all exceeding 0.920. Notably, the significant improvement in the recall metric for sparse seedling canopies ensures reliable data extraction during the critical initial phase of canopy closure dynamics. These results are consistent with previous studies where DeepLabv3+ has shown excellent coverage recognition for maize leaves, rice panicles, and trees [28,29,30].

The semantic segmentation models selected for this study, such as DeepLabv3+ and U-Net, have demonstrated robust and reliable performance. They provide strong support for rice canopy coverage extraction and canopy closure dynamic analysis. However, there remains a high scope of rapid development in technological innovation and iteration within the field of deep learning. There is a continuous emergence of new model architectures and optimization strategies, which can provide further improvements in segmentation precision. Therefore, future research should prioritize higher segmentation accuracy as a core objective. A key direction for future work is the substantial expansion and enhancement of current datasets by incorporating a more diverse range of rice genetic backgrounds, differentiated ecological and environmental conditions, and more comprehensive data that can cover all growth stages throughout the entire life cycle. These datasets can provide more holistic feature information that can be useful for model training, thereby improving both the generalization capability and precision of segmentation models in complex scenarios.

### 3.3. Canopy Closure Dynamics and Rice Yield

**Figure 8:**
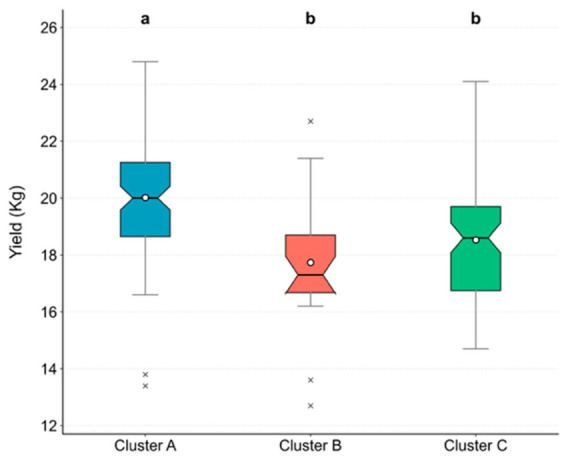
Comparison of grain yield among the three identified rice clusters. Different letters above the boxplots indicate significant differences at the *p* < 0.05 level based on Tukey’s HSD test.

Previous studies have demonstrated a significant correlation between crop canopy coverage and final yield [31,32]. In this study, the 198 rice varieties were categorized into three distinct clusters based on five core dynamic indicators. As illustrated in Figure 8, Cluster A achieved a significantly higher grain yield than Clusters B and C (*p* < 0.05). While the yield difference between Cluster B and Cluster C was not statistically significant (both marked with the letter ‘b’), the mean yield of Cluster C was numerically higher than that of Cluster B.

According to Figure 6, although no significant difference was observed in the *K* values across clusters, all other dynamic indicators exhibited highly significant differences. Cluster A was characterized by the highest *MGR* and the shortest *Tm*, leading to rapid early-stage canopy expansion and superior yield performance. Conversely, Cluster B featured the lowest *MGR* and the longest *Tm*, representing a conservative growth rhythm with the slowest early-stage coverage expansion and the lowest yield. Cluster C exhibited intermediate values for *a*, *MGR*, and *Tm*; although its growth rhythm was more balanced than the conservative pattern of Cluster B, these moderate phenotypic advantages did not translate into a statistically significant yield increase, though they contributed to a higher mean yield relative to Cluster B.

**Figure 9:**
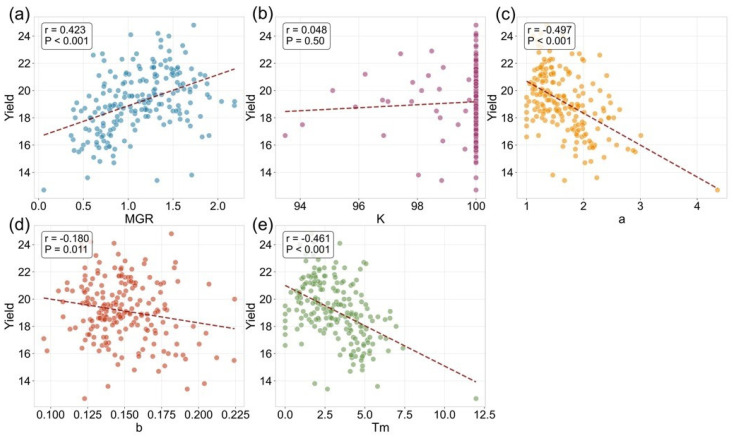
Correlation analysis between rice yield and canopy closure dynamic indicators: (**a**) *MGR* indicator; (**b**) *K* indicator; (**c**) *a* indicator; (**d**) *b* indicator; (**e**) *Tm* indicator.

Pearson correlation analysis was used to explore the relationship between rice yield and dynamic indicators. As Figure 9 shows, *MGR* exhibited a highly significant positive correlation with yield (*r* = 0.423, *p* < 0.001). This strong association indicates that a faster early growth rate provides a more robust biomass foundation, thereby strongly supporting yield formation. Conversely, *a* and *Tm* showed significant negative correlations with yield (*r* = −0.497, *p* < 0.001 and *r* = −0.461, *p* < 0.001, respectively), which implies that a smaller *a* value and an earlier occurrence of the growth peak allow the crop to establish stable growth sooner. Consequently, the impact of late-stage nutrient competition and environmental stress is reduced, which increases the yield potential. Furthermore, *b* showed a weak negative correlation with yield (*r* = −0.180, *p* = 0.011), signifying a slight inverse regulatory effect, while *K* exhibited no significant linear correlation, and therefore is not considered a core factor influencing yield. This indicates that rice yield formation is not determined by the final canopy coverage level but depends more on the canopy closure rhythm and rate during early growth stages. These results closely match the physiological patterns of rice growth and development.

The tillering stage is a critical phase for the establishment of population structure. Varieties that achieve early canopy closure with high growth efficiency can form an optimal leaf area index during peak tillering, which ensures a rational distribution of light between the upper and lower canopy layers and enhances the population photosynthetic rate. Simultaneously, sufficient early vegetative growth promotes the formation of effective tillers and increases the number of panicles per unit area. This results in increased yield, as the number of effective panicles is a core component of rice yield and directly determines its yield potential [33,34,35]. However, it is important to note that the positive associations observed here represent correlations specific to the environmental conditions of the tested season. Further multi-environment trials are necessary to validate the broader agronomic applicability of these findings.

Rice canopy closure dynamics are closely related to yield formation, as they regulate the rhythm of population structure construction and resource utilization efficiency. The positive correlation between early canopy closure and high efficiency growth characteristics and high yield provides empirical data support for the synergy between dynamic growth and yield in rice variety screening and planting strategy optimization. Ultimately, this offers a vital theoretical basis for the construction of high-yield and high-efficiency rice cultivation models.

Translating these theoretical insights into agronomic practice, the classification of rice varieties into distinct dynamic clusters offers profound implications for field phenotyping, shifting the focus from static final traits to dynamic early-growth rhythms. From a breeding perspective, dynamic indicators such as a high *MGR* and short *Tm* can serve as reliable early-stage phenotypic biomarkers. This enables breeders to efficiently identify high-yielding genotypes (e.g., Cluster A) within the first month after transplantation, significantly accelerating the screening process. From a cultivation perspective, these cluster-specific growth trajectories provide actionable insights for precision agriculture. For instance, the rapid early canopy closure observed in Cluster A varieties presents a significant ecological advantage by swiftly shading the soil surface. This rapid shading strongly suppresses early weed emergence, thereby reducing reliance on chemical herbicides. Agronomically, Cluster A varieties require sufficient basal fertilizer to support their vigorous early growth, followed by timely mid-season drainage to mitigate the risk of lodging. In contrast, the slow-starting varieties in Cluster B may benefit from higher initial planting densities or targeted mid-tillering nitrogen topdressing to compensate for their delayed canopy expansion. Ultimately, quantifying these dynamic differences allows agronomists to tailor water, fertilizer, and density management strategies to the specific biological characteristics of each variety.

### 3.4. Limitations and Future Perspectives

This study establishes a comprehensive framework for monitoring and analyzing rice canopy closure dynamics; however, several limitations warrant acknowledgment. First, the experimental setting and environmental conditions were relatively homogeneous. Variations in climate, soil properties, and cropping systems across diverse ecological zones may affect the robustness of the results, potentially limiting their direct generalizability to other rice-growing regions [36]. Second, the experimental duration and data dimensions were constrained. The current dataset only covered a single growing season, without accounting for interannual climate fluctuations. Consequently, the results lack multi-year validation and do not integrate influential factors such as soil physicochemical properties or specific water and fertilizer management regimes [37]. Third, the study reflects limited application scenarios and sample diversity. The training samples shared a relatively narrow genetic background and excluded imagery data from extreme growth conditions, which may restrict model generalization. Furthermore, the Gompertz model exhibited sub-optimal fitting performance for certain varieties displaying atypical growth patterns. These atypical trajectories were primarily observed in plots experiencing localized micro-environmental stress, such as uneven water distribution, or in specific varieties with highly erect leaf architectures that significantly delayed the visual canopy closure phase. Such patterns deviate from the standard sigmoidal growth curve inherent to the Gompertz model, aligning with previous findings that suggest extreme traits can compromise fitting precision [38]. Fourth, the depth of the indicator system and mechanistic analysis remains insufficient. Core dynamic indicators were derived solely from canopy coverage, overlooking physiological, biochemical, or three-dimensional structural parameters. Therefore, establishing the intrinsic physiological links between canopy closure dynamics and final yield requires further investigation [39].

Considering these limitations and the emerging trends toward precision and intelligent agriculture, future research should focus on the following aspects. The experimental scope should be expanded, and the monitoring period extended. Multi-site trials should be conducted across diverse rice ecological zones and include a wide range of rice varieties to construct a comprehensive multi-region, multi-variety, and multi-year dataset. Such efforts would enhance the generalizability and stability of the results [40]. Data dimensionality should be enriched and model performance further optimized. Model adaptability to complex and heterogeneous scenarios can be improved by integrating UAV-based hyperspectral and LiDAR data with physiological, biochemical, and soil parameters, as well as by introducing advanced deep learning techniques, such as Transformer architectures and transfer learning strategies [41,42].

Furthermore, deeper mechanistic research should be conducted and the indicator system further refined. Multidisciplinary approaches should be adopted to investigate the relationships between dynamic indicators and rice physiological traits. In addition, the indicator set should be expanded to incorporate parameters related to three-dimensional canopy structure and stress resistance, thereby establishing a more comprehensive evaluation framework [43]. Finally, technology transfer and practical application should be actively promoted. The proposed technical framework should be integrated with crop growth models to develop yield prediction and cultivation decision-support systems. Furthermore, the development of lightweight field monitoring software would facilitate the practical deployment of this system in grassroots agricultural extension and variety screening by seed enterprises [44].

## 4. Materials and Methods

### 4.1. Field Experiments and Materials

**Figure 10:**
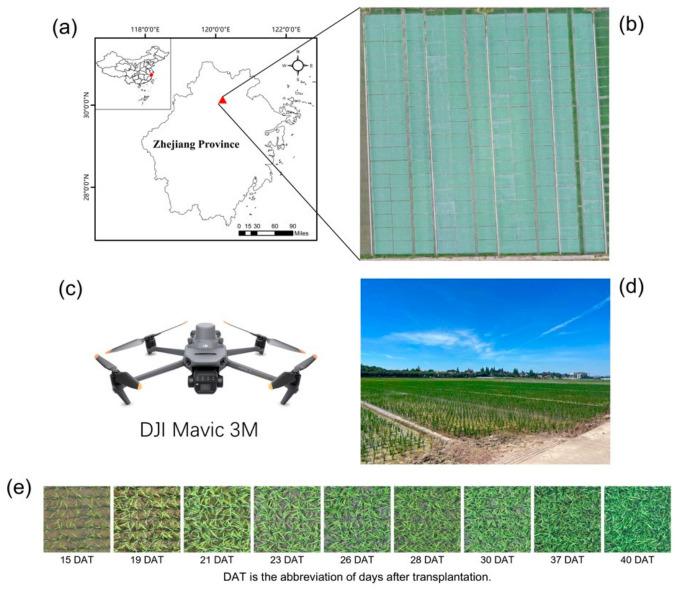
Overview of the experimental site and data acquisition. (**a**) Geographical location of the study area; (**b**) spatial layout of the 198 experimental rice plots; (**c**) the DJI Mavic 3M RTK UAV platform used for imagery collection; (**d**) ground view of the experimental farmland; (**e**) multi-temporal evolution of canopy coverage within a representative plot across different growth stages.

The experiment was conducted at the Yangdu Research and Innovation Base of the Zhejiang Academy of Agricultural Sciences in Jiaxing, Zhejiang Province, China (120°24′ E, 30°26′ N). The study area features a subtropical monsoon climate, with an average annual rainfall of 1400 mm and a mean daily temperature of 16.8 °C. The location of the study area and the experimental plots are shown in Figure 10a and b, respectively. The experimental field comprised 198 plots, each measuring 4 × 5 m. A total of 198 hybrid combinations, derived from 47 *japonica* rice populations via incomplete diallel crosses, were evaluated. Each combination was transplanted into a separate plot following a standard planting configuration of 14 rows and 24 columns of seedlings, totaling 336 hills per plot. This configuration resulted in a plant population density of 16.8 hills/m^2^. All plots received identical nitrogen fertilization and irrigation management. Rice was sown on 20 May 2025, and seedlings were transplanted on 18 June 2025; harvesting was subsequently conducted on 30 October 2025. To determine the final yield for each variety, the total weight of husked rice from the entire plot was recorded. Notably, because the experimental plots were arranged contiguously and a dedicated buffer zone of protective rice rows was established around the entire research perimeter to isolate environmental border effects, the marginal effects on individual plots were minimized. Consequently, yield data were collected from the whole plot area rather than excluding edge rows, ensuring a high-throughput and consistent sampling process across all 198 combinations.

### 4.2. Image Data Acquisition

As illustrated in Figure 10c, a DJI Mavic 3M RTK UAV (DJI Technology, Shenzhen, China) was utilized to capture RGB images with a sensor resolution of 5280 × 3956 pixels. Data collection was conducted over the experimental plots shown in Figure 10b, focusing on the early growth stages of rice from 15 to 40 days after transplantation (DAT). A total of nine flight operations were executed between 10:00 AM and 12:00 PM on clear days, spanning the seedling to jointing stages (Figure 10e). The UAV followed predetermined flight paths at a constant altitude of 16 m and a speed of 5 m/s, with longitudinal and lateral overlaps sufficient for high-quality orthomosaic generation. Table 4 summarizes the planting scheme and the specific data acquisition calendar. The raw images were subsequently processed using DJI Terra 5.1.1 software to generate georeferenced orthomosaics for further analysis.

### 4.3. Data Preprocessing

The generated orthomosaics underwent a systematic preprocessing workflow to construct a high-quality dataset for semantic segmentation training. First, a Python-based image processing library (PIL) was utilized to crop the orthomosaics into 512 × 512 pixel patches. To ensure these patches were accurately aligned with the experimental plots and excluded non-target areas, a standardized grid-based cropping workflow was implemented. Specifically, the central region of each plot was identified using GIS-based shapefiles that defined the plot boundaries. Image patches were then extracted strictly from these specific coordinates to represent individual varieties, effectively eliminating the gaps between plots and ensuring that each patch contained only the target rice canopy. Second, a rigorous quality screening process was conducted at the patch level to ensure dataset consistency and illumination stability. Image patches exhibiting severe cloud shadows, excessive specular reflection from the paddy water surface, or motion blur induced by strong winds were manually inspected and excluded. This two-step approach combining GIS-based spatial filtering with manual quality control guaranteed that only high-quality representative data entered the training pipeline. Third, the interactive semi-automatic annotation tool based on the Segment Anything Model (ISAT-SAM) was utilized to manually annotate the rice canopy areas as a single class. In total, 314 high-quality images were annotated, comprehensively covering the seedling, tillering, and jointing stages to capture a full spectrum of canopy density variations. Finally, the annotated dataset was randomly divided into a training set and a validation set at a 7:3 ratio at the plot level. To ensure objective evaluation, all image patches derived from the exact same plot on the same date were exclusively assigned to either the training or the validation set, ensuring no cross-contamination. The 70% training subset was used for the iterative optimization of model parameters, while the remaining 30% served as the validation set to objectively evaluate the models’ generalization ability.

### 4.4. Rice Canopy Segmentation Methods

This study proposes an accurate system for rice canopy coverage prediction, which is based on three currently used popular models that have performed well in the semantic segmentation field: DeepLabv3+, U-Net, and PSPNet.

The multi-scale sampling mechanism of atrous convolution in DeepLabv3+ [45] allows it to flexibly adapt to the variations in canopy density that occur between the seedling stage to maturity. In sparse canopy scenarios, its encoder–decoder structure effectively preserves detailed features such as leaf edges and scattered tillers, which prevent omissions caused by low canopy coverage and small target pixel proportions. The adjustment of dilation rates in atrous convolutions during the canopy closure stage allows for the capture of overall canopy contours and hierarchical relationships that reduce segmentation blurring in overlapping leaf areas. This adaptability to varying coverage scenarios makes DeepLabv3+ stand out in recall metrics, particularly for application scenarios that require precise statistics of actual canopy coverage.

The U-Net [46] focuses on adaptability to small sample sizes and complex backgrounds. Rice field imagery often contains interference caused by factors such as soil texture variations, uneven weed distribution, and light fluctuations. Furthermore, practical research may face high sample collection costs as well as limited data availability. The advantage of U-Net’s symmetric contraction–expansion path design lies in the contraction path that can extract high-level semantic features of the canopy through downsampling. On the other hand, the expansion path fuses low-level detailed features through upsampling and skip connections. This feature fusion mechanism enables U-Net to precisely differentiate between the rice canopy and background noise even under small-sample training. It can avoid misclassifying weeds or stubble as canopy while completely capturing the continuous canopy distribution, especially in medium-coverage scenarios. The high-resolution masks provided by U-Net accurately reflect the spatial heterogeneity of canopy coverage and provide reliable support for quantitative calculations.

PSPNet [47] enables rice canopy coverage assessment by precisely identifying individual leaf areas and the overall distribution pattern of the canopy. Its pyramid pooling module combines local leaf features with global canopy distribution characteristics through pooling operations at different scales. This combination effectively avoids local over-segmentation in high-density canopy scenarios caused by dense leaf overlapping, which results in accurate delineation of the boundaries between canopy and non-canopy areas and a reduction in false-positive labeling. Simultaneously, its global feature extraction capability allows it to ignore sporadically distributed non-canopy interference and focus on the overall coverage range. This capacity to extract global coverage patterns provides excellent precision, making it suitable for macro-statistics of canopy coverage where there is a low misclassification tolerance.

Semantic segmentation models were implemented using the PyTorch 2.9.0 framework. For all three architectures (DeepLabv3+, U-Net, and PSPNet), MobileNetV2 was utilized as the feature extraction backbone, with pre-trained weights loaded to accelerate convergence. To enhance model robustness and prevent overfitting, data augmentation strategies such as random horizontal flipping and image scaling (0.5 to 1.5) were applied during training. All models were trained for 100 epochs using a two-stage transfer learning strategy. The backbone was frozen for the first 50 epochs with a batch size of 8 and subsequently unfrozen for the remaining 50 epochs with a batch size of 4. Model parameters were optimized using the stochastic gradient descent (SGD) algorithm with a momentum of 0.9 and a weight decay of 0.0001. The standard cross-entropy loss function was employed. The initial learning rate was set to 0.007 and progressively reduced using a cosine annealing scheduler. Computational tasks were executed on a workstation equipped with an Intel Core i7-12700 CPU and an NVIDIA GeForce RTX 3080 Ti GPU (12 GB VRAM).

### 4.5. Metrics for Canopy Segmentation Accuracy Evaluation

Five core evaluation metrics were introduced to avoid model overfitting caused by data partition bias and to comprehensively evaluate the segmentation performance: intersection over union (IoU), pixel accuracy (PA), precision, recall, and F1-score. Out of these, the IoU serves as the primary metric for segmentation accuracy assessment. It reflects the degree of overlap between the predicted canopy mask and the ground truth as it is calculated as a ratio of their intersection and union. PA represents the overall classification precision and measures the proportion of correctly classified pixels at the pixel level. Precision characterizes a model’s ability to avoid misclassifying non-canopy regions, such as soil, weeds, and water, as rice canopy, while recall ensures that sparse canopy areas can be identified effectively, preventing omissions caused by low canopy coverage. Finally, the F1-score is calculated as the harmonic mean of precision and recall. It balances the performance differences between precision and recall and effectively mitigates the limitations of aforementioned single-indicator assessments.

The chosen evaluation metrics are particularly suitable for comparing the comprehensive segmentation performance of different models in complex field scenarios, and thus provide a scientific basis for selecting the optimal prediction model for rice canopy coverage. The aforementioned metrics are calculated as follows:

(1) $$IoU\;=\;\frac{TP}{TP\;+\;FP\;+\;FN}$$

(2) $$PA\;=\frac{TP+TN}{TP+TN+FP+FN}$$

(3) $$Precision=\frac{TP}{TP+FP}$$

(4) $$Recall=\frac{TP}{TP+FN}$$

(5) $$F1\;score=2\frac{Precision\;\times \;Recall}{Precision\;+Recall}$$

In the above expressions, True positive (*TP*) represents the number of correctly identified canopy pixels; True negative (*TN*) represents the number of correctly identified background pixels; False positive (*FP*) represents the number of background pixels that are incorrectly identified as canopy; and False negative (*FN*) represents the number of canopy pixels that are incorrectly identified as background. The mean values of the five aforementioned indicators are utilized to compare model performance more objectively, where these values are denoted by mIoU, mPA, mPrecision, mRecall, and Macro-F1. These metrics provide a comprehensive evaluation of the overall segmentation performance of the models.

### 4.6. Rice Canopy Closure Dynamic Analysis

**Figure 11:**
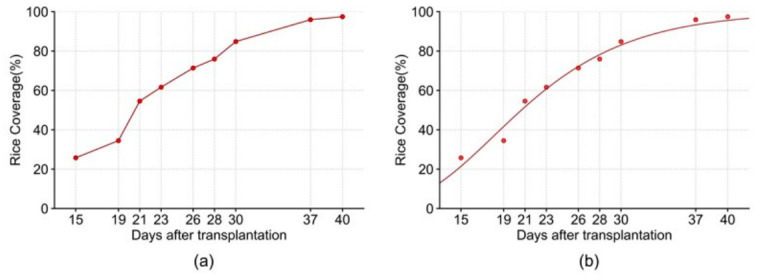
(**a**) Dynamic change curve of rice coverage. (**b**) Gompertz model fitting curve.

The canopy closure trends of different rice varieties were extracted based on the semantic segmentation results. The dynamic changes in canopy closure were described using two types of descriptive indicators, which included nine time-series indicators and five growth characteristic indicators. Figure 11a illustrates the dynamic change curve of rice coverage. It was observed that between relative time points 0 and 25, the rice canopy coverage increased slowly during the seedling stage, then increased rapidly during the tillering stage, and finally stabilized during the jointing stage.

The Gompertz model is a classic nonlinear model for characterizing crop growth dynamics, which offers higher fitting precision for rate changes around the growth inflection point compared to models such as Logistic and Richards. It is particularly suitable for mathematically capturing the biological “slow start, rapid growth, plateau” curve of actual crop populations [48,49]. By introducing three core parameters, i.e., the canopy closure limit value, the initial growth coefficient, and the growth rate coefficient, this model can both precisely capture the differences in growth potential among various rice varieties and quantify the variety-specific growth rhythms. Therefore, this study selected the Gompertz model to fit the time-series data of canopy coverage for each plot, and the corresponding fitted curve of the Gompertz model is shown in Figure 11b. The coverage is calculated using the Gompertz model as follows:

(6) $$V(t)\;=Ke^{-ae^{-bt}}$$

Five dynamic indicators were extracted after fitting. (1) Canopy closure limit (*K*), which reflects the maximum population coverage degree that a specific variety can achieve during its growth cycle. It serves as the core metric for assessing the sufficiency of canopy closure. (2) Initial growth coefficient (*a*), which reflects the resistance or inertia in the early growth stage of rice and is a key parameter for early growth posture. (3) Growth rate coefficient (*b*), which reflects the rice coverage growth intensity and efficiency, and is directly related to the coverage growth rhythm during the growth cycle. (4) Maximum instantaneous growth rate (*MGR*), which describes the maximum value of the instantaneous growth rate. It directly reflects the population expansion ability of a variety during its vigorous growth period and serves as the core indicator for quantifying growth vitality. (5) Days to maximum growth rate (*Tm*), which refers to the time interval from the start of growth to reaching the *MGR* and provides a direct reflection of the timing, i.e., early or late rhythm of the peak growth period. The *MGR* and *Tm* are calculated based on the following expressions:

(7) $$MGR=Kbe^{-(a+1)}$$

(8) $$T_{m}=\frac{ln(a)}{b}\;(a>0)$$

Three statistical indicators were further introduced to evaluate the fitting performance: the coefficient of determination (R^2^), root mean square error (RMSE), and mean absolute error (MAE). Out of these, R^2^ reflects the degree of fit between the fitted curve and the measured data, while RMSE and MAE quantify the deviation between them.

### 4.7. Classification of Rice Varieties Based on Canopy Closure Dynamics

This study employed K-means clustering analysis to categorize the tested rice varieties into distinct groups. This categorization is used to quantitatively highlight the differences in rice canopy closure characteristics among various rice varieties. The K-means clustering [50] is a classic algorithm in the field of unsupervised machine learning that has been widely applied across multidisciplinary data classification research. It establishes similarity metrics based on the Euclidean distance between samples in a multidimensional data space. Under a specified fixed number of clusters, it iteratively optimizes the clustering process to minimize the sum of squared distances from samples within each cluster to their respective centroids. Ultimately, automated classification of multiple clusters is realized by assigning samples that are closer to a specific centroid to the same group. The K-means clustering algorithm offers significant advantages compared to traditional classification methods or other clustering algorithms, including high objectivity, rigorous mathematical logic, and robust generalization capabilities.

To characterize rice canopy closure dynamics, five indicators were selected as input variables for the K-means model. Table 5 shows the significance of these five indicators. The elbow method and silhouette coefficient [51] were utilized to determine the optimal number of clusters, which was identified as three. Following the classification of the 198 varieties, analysis of variance (ANOVA) and Tukey’s HSD test (*p* < 0.05) were performed to assess the differences in dynamic indicators among the clusters. All statistical procedures, including K-means clustering and one-way ANOVA, were executed using the “stats” and “cluster” packages in R 4.5.0 software.

## 5. Conclusions

This study established an efficient and precise monitoring system for rice canopy closure dynamics by integrating UAV remote sensing with deep learning algorithms. The results demonstrated that the DeepLabv3+ model outperformed U-Net and PSPNet, providing a robust data foundation for high-throughput phenotyping. By employing the Gompertz model to fit time-series coverage data, we successfully extracted key dynamic indicators that captured the nuances of rice growth rhythms across 198 varieties. The subsequent clustering analysis revealed that the primary distinctions among rice varieties lie in their early-stage growth rates rather than their final coverage levels. Furthermore, a significant correlation was observed between early canopy closure dynamics and grain yield. Specifically, varieties in Cluster A, characterized by rapid early-season growth, achieved higher yields, suggesting that early-stage growth acceleration is a promising indicator for yield potential. However, it is important to note that these findings are based on data collected from a single experimental site during a single growing season. While the observed relationships are statistically robust within this context, they should be interpreted as strong evidence of correlation rather than universally applicable agronomic rules. The specific model parameters and growth thresholds may be subject to genotype-by-environment (G × E) interactions. Future research should involve multi-year and multi-site trials to validate the stability of these indicators across diverse agro-ecological conditions. Nevertheless, the methodological framework developed in this study provides a scalable and objective tool for large-scale rice breeding and precision field management.

## Figures and Tables

**Table 1:** Performance comparison of semantic segmentation models for rice canopy segmentation.

Model	mIoU	mPA	mPrecision	mRecall	Macro-F1
DeepLabv3+	0.860	0.925	0.922	0.925	0.924
PSPNet	0.647	0.766	0.789	0.766	0.776
U-Net	0.845	0.912	0.917	0.912	0.914

**Table 2:** DeepLabv3+ model’s segmentation performance across different growth stages.

Stage Code	Growth Stage	Coverage	mIoU	mPA	mPrecision	mRecall	Macro-F1
GS1	Seedling	32.6%	0.782	0.895	0.901	0.853	0.876
GS2	Seedling	41.8%	0.807	0.903	0.912	0.878	0.894
GS3	Tillering	58.5%	0.861	0.928	0.935	0.926	0.930
GS4	Tillering	65.2%	0.873	0.932	0.938	0.931	0.934
GS5	Tillering	76.4%	0.885	0.941	0.943	0.939	0.941
GS6	Tillering	84.7%	0.890	0.945	0.946	0.944	0.945
GS7	Tillering	88.3%	0.887	0.943	0.942	0.943	0.942
GS8	Tillering	92.1%	0.879	0.938	0.936	0.937	0.936
GS9	Jointing	95.8%	0.851	0.922	0.920	0.910	0.915

**Table 3:** The fitting results of the Gompertz model.

Statistical Index	Mean	Minimum	Maximum	Standard Deviation
R^2^	0.978	0.952	0.994	0.011
RMSE	2.36%	1.21%	3.87%	0.58%
MAE	1.89%	0.95%	3.02%	0.45%

**Table 4:** Planting scheme and data acquisition calendar.

Growth Stage Code	Date	Growth Stage	Days After Transplantation (DAT)
GS1	3 July 2025	Seedling	15
GS2	7 July 2025	Seedling	19
GS3	9 July 2025	Tillering	21
GS4	11 July 2025	Tillering	23
GS5	14 July 2025	Tillering	26
GS6	16 July 2025	Tillering	28
GS7	18 July 2025	Tillering	30
GS8	25 July 2025	Tillering	37
GS9	28 July 2025	Jointing	40

**Table 5:** Significance of rice coverage dynamic indicators used for K-means clustering analysis of rice varieties.

Indicator	Meaning
*K*	Upper threshold of canopy coverage
*a*	Threshold for early growth initiation
*b*	Acceleration capacity of coverage growth
*MGR*	Peak rate during canopy closure
*Tm*	Timing of maximum growth rate

## Data Availability

The data presented in this study are available on request from the corresponding author due to privacy.

## Abbreviations

The following abbreviations are used in this manuscript:

UAV	Unmanned aerial vehicle
IoU	Intersection over union
DAT	Days after transplantation
GS	Growth stage
PA	Pixel accuracy
RMSE	Root mean square error
MAE	Mean absolute error

## References

[1] Xu L., Yuan S., Man J.G. (2020). Changes in rice yield and yield stability in China during the past six decades. J. Sci. Food Agric..

[2] Nakano S., Fujii N., Koyama R., Uno Y. (2025). Prediction of Lettuce Harvest Date and Evaluation of Data for Yield Estimation Using Artificial Intelligence Analysis of Aerial Drone Images. Hortic. J..

[3] Maimaitijiang M., Ghulam A., Sidike P., Hartling S., Maimaitiyiming M., Peterson K., Shavers E., Fishman J., Peterson J., Kadam S. (2017). Unmanned Aerial System (UAS)-based phenotyping of soybean using multi-sensor data fusion and extreme learning machine. ISPRS-J. Photogramm. Remote Sens..

[4] Han L., Yang G.J., Yang H., Xu B., Li Z.H., Yang X.D. (2018). Clustering Field-Based Maize Phenotyping of Plant-Height Growth and Canopy Spectral Dynamics Using a UAV Remote-Sensing Approach. Front. Plant Sci..

[5] Maimaitijiang M., Sagan V., Sidike P., Maimaitiyiming M., Hartling S., Peterson K.T., Maw M.J.W., Shakoor N., Mockler T., Fritschi F.B. (2019). Vegetation Index Weighted Canopy Volume Model (CVMVI) for soybean biomass estimation from Unmanned Aerial System-based RGB imagery. ISPRS-J. Photogramm. Remote Sens..

[6] Reza M.N., Na I.S., Lee K.H. (2017). Automatic Counting of Rice Plant Numbers After Transplanting Using Low Altitude UAV Images. Int. J. Contents.

[7] Vayssade J.A., Paoli J.N., Gée C., Jones G. (2021). DeepIndices: Remote Sensing Indices Based on Approximation of Functions through Deep-Learning, Application to Uncalibrated Vegetation Images. Remote Sens..

[8] Jin X.J., Han K., Zhao H., Wang Y., Chen Y., Yu J.L. (2024). Detection and coverage estimation of purple nutsedge in turf with image classification neural networks. Pest Manag. Sci..

[9] Tanabe R., Matsui T., Tanaka T.S.T. (2023). Winter wheat yield prediction using convolutional neural networks and UAV-based multispectral imagery. Field Crop. Res..

[10] Bai Y., Shi L.S., Zha Y.Y., Liu S.B., Nie C.W., Xu H.G., Yang H.Y., Shao M.C., Yu X., Cheng M.H. (2023). Estimating leaf age of maize seedlings using UAV-based RGB and multispectral images. Comput. Electron. Agric..

[11] Sato Y., Tsuji T., Matsuoka M. (2024). Estimation of Rice Plant Coverage Using Sentinel-2 Based on UAV-Observed Data. Remote Sens..

[12] Xie Z.W., Chen S., Gao G.Z., Li H., Wu X.M., Meng L., Ma Y.T. (2022). Evaluation of rapeseed flowering dynamics for different genotypes with UAV platform and machine learning algorithm. Precis. Agric..

[13] Zhang D.H., Hou L., Lv L.J., Qi H., Sun H.F., Zhang X.S., Li S., Min J.A., Liu Y.W., Tang Y.Y. (2025). Precision Agriculture: Temporal and Spatial Modeling of Wheat Canopy Spectral Characteristics. Agriculture.

[14] Sadras V.O., Rebetzke G.J., Edmeades G.O. (2013). The phenotype and the components of phenotypic variance of crop traits. Field Crop. Res..

[15] Furbank R.T., Jimenez-Berni J.A., George-Jaeggli B., Potgieter A.B., Deery D.M. (2019). Field crop phenomics: Enabling breeding for radiation use efficiency and biomass in cereal crops. New Phytol..

[16] Grosskinsky D.K., Svensgaard J., Christensen S., Roitsch T. (2015). Plant phenomics and the need for physiological phenotyping across scales to narrow the genotype-to-phenotype knowledge gap. J. Exp. Bot..

[17] Herwitz S., Johnson L., Dunagan S., Higgins R., Sullivan D., Zheng J., Lobitz B., Leung J., Gallmeyer B., Aoyagi M. (2004). Imaging from an unmanned aerial vehicle: Agricultural surveillance and decision support. Comput. Electron. Agric..

[18] Bongomin O., Lamo J., Guina J.M., Okello C., Ocen G.G., Obura M., Alibu S., Owino C.A., Akwero A., Ojok S. (2024). UAV image acquisition and processing for high-throughput phenotyping in agricultural research and breeding programs. Plant Phenome J..

[19] Impollonia G., Croci M., Ferrarini A., Brook J., Martani E., Blandinières H., Marcone A., Awty-Carroll D., Ashman C., Kam J. (2022). UAV Remote Sensing for High-Throughput Phenotyping and for Yield Prediction of Miscanthus by Machine Learning Techniques. Remote Sens..

[20] Rasmussen J., Ntakos G., Nielsen J., Svensgaard J., Poulsen R.N., Christensen S. (2016). Are vegetation indices derived from consumer-grade cameras mounted on UAVs sufficiently reliable for assessing experimental plots?. Eur. J. Agron..

[21] Du M.M., Noguchi N. (2017). Monitoring of Wheat Growth Status and Mapping of Wheat Yield’s within-Field Spatial Variations Using Color Images Acquired from UAV-camera System. Remote Sens..

[22] Duan T., Chapman S.C., Guo Y., Zheng B. (2017). Dynamic monitoring of NDVI in wheat agronomy and breeding trials using an unmanned aerial vehicle. Field Crop. Res..

[23] Wang Z.H., Skidmore A.K., Darvishzadeh R., Wang T.J. (2018). Mapping forest canopy nitrogen content by inversion of coupled leaf-canopy radiative transfer models from airborne hyperspectral imagery. Agric. For. Meteorol..

[24] Cen H.Y., Wan L., Zhu J.P., Li Y.J., Li X.R., Zhu Y.M., Weng H.Y., Wu W.K., Yin W.X., Xu C. (2019). Dynamic monitoring of biomass of rice under different nitrogen treatments using a lightweight UAV with dual image-frame snapshot cameras. Plant Methods.

[25] Liu W.W., Mottus M., Gastellu-Etchegorry J.P., Fang H.L., Atherton J. (2024). Seasonal and vertical variation in canopy structure and leaf spectral properties determine the canopy reflectance of a rice field. Agric. For. Meteorol..

[26] Sun B.F., Li Y.D., Huang J.B., Cao Z.S., Peng X.Y. (2024). Impacts of Variable Illumination and Image Background on Rice LAI Estimation Based on UAV RGB-Derived Color Indices. Appl. Sci..

[27] Li C.M., Teng X., Tan Y., Zhang Y., Zhang H.C., Xiao D., Luo S.J. (2024). Spatio-temporal mapping of leaf area index in rice: Spectral indices and multi-scale texture comparison derived from different sensors. Front. Plant Sci..

[28] Bak H.J., Kim E.J., Lee J.H., Chang S.Y., Kwon D., Im W.J., Hwang W.H., Chang J.K., Chung N.J., Sang W.G. (2025). Deep learning-based semantic segmentation for rice yield estimation by analyzing the dynamic change of panicle coverage. Front. Plant Sci..

[29] Zhou C.Q., Ye H.B., Xu Z.F., Hu J., Shi X.Y., Hua S., Yue J.B., Yang G.J. (2019). Estimating Maize-Leaf Coverage in Field Conditions by Applying a Machine Learning Algorithm to UAV Remote Sensing Images. Appl. Sci..

[30] Popp M.R., Kalwij J.M. (2023). Consumer-grade UAV imagery facilitates semantic segmentation of species-rich savanna tree layers. Sci. Rep..

[31] Wan L., Li Y.J., Cen H.Y., Zhu J.P., Yin W.X., Wu W.K., Zhu H.Y., Sun D.W., Zhou W.J., He Y. (2018). Combining UAV-Based Vegetation Indices and Image Classification to Estimate Flower Number in Oilseed Rape. Remote Sens..

[32] Wan L., Cen H.Y., Zhu J.P., Zhang J.F., Zhu Y.M., Sun D.W., Du X.Y., Zhai L., Weng H.Y., Li Y.J. (2020). Grain yield prediction of rice using multi-temporal UAV-based RGB and multispectral images and model transfer—A case study of small farmlands in the South of China. Agric. For. Meteorol..

[33] Pan Y., Cao Y., Chai Y., Meng X., Wang M., Wang G., Guo S. (2023). Identification of photosynthetic parameters for superior yield of two super hybrid rice varieties: A cross-scale study from leaf to canopy. Front. Plant Sci..

[34] Zhang J., Zhang Y., Chen J., Xu M., Guan X., Wu C., Zhang S., Qu H., Chu J., Xu Y. (2024). Sugar transporter modulates nitrogen-determined tillering and yield formation in rice. Nat. Commun..

[35] Yang X.L., Wang X.X., Li Y., Yang L.T., Hu L., Han Y.L., Wang B.F. (2024). Effects of Drought Stress at the Booting Stage on Leaf Physiological Characteristics and Yield of Rice. Plants.

[36] Wang S.Y., Liu Y.J., Asseng S., Harrison M.T., Tang L., Liu B., Liu K., Luo Z.K., Wang E.L., Chang J.F. (2025). Rice yield stability and its determinants across different rice-cropping systems in China. Agric. For. Meteorol..

[37] Chen H., Wu Y.C., Cheng C.C., Teng C.Y. (2023). Effect of climate change-induced water-deficit stress on long-term rice yield. PLoS ONE.

[38] Korkmaz M. (2021). A study over with four-parameter Logistic and Gompertz growth models. Numer. Methods Partial. Differ. Equ..

[39] Kakar N., Bheemanahalli R., Jumaa S., Redoña E., Warburton M.L., Reddy K.R. (2021). Assessment of agro-morphological, physiological and yield traits diversity among tropical rice. PeerJ.

[40] Dingkuhn M., Sow A., Manneh B., Radanielina T., Raboin L.M., Dusserre J., Ramantsoanirina A., Shrestha S., Ahmadi N., Courtois B. (2015). Field phenomics for response of a rice diversity panel to ten environments in Senegal and Madagascar. 1. Plant phenological traits. Field Crop. Res..

[41] Sahoo R.N., Rejith R.G., Gakhar S., Verrelst J., Ranjan R., Kondraju T., Meena M.C., Mukherjee J., Dass A., Kumar S. (2024). Estimation of wheat biophysical variables through UAV hyperspectral remote sensing using machine learning and radiative transfer models. Comput. Electron. Agric..

[42] Ulukaya S., Deari S. (2025). A robust vision transformer-based approach for classification of labeled rices in the wild. Comput. Electron. Agric..

[43] Chen L., Deng X.Y., Duan H.X., Tan X.M., Xie X.B., Pan X.H., Guo L., Luo T., Chen X.B., Gao H. (2025). Canopy humidity and irrigation regimes interactively affect rice physiology, grain filling and yield during grain filling period. Agric. Water Manag..

[44] Zheng H.W., Liu C.J., Zhong L., Wang J., Huang J.M., Lin F., Ma X., Tan S.Y. (2025). An android-smartphone application for rice panicle detection and rice growth stage recognition using a lightweight YOLO network. Front. Plant Sci..

[45] Chen L.C., Papandreou G., Kokkinos I., Murphy K., Yuille A.L. (2018). DeepLab: Semantic Image Segmentation with Deep Convolutional Nets, Atrous Convolution, and Fully Connected CRFs. IEEE Trans. Pattern Anal. Mach. Intell..

[46] Ronneberger O., Fischer P., Brox T. (2015). U-Net: Convolutional Networks for Biomedical Image Segmentation.

[47] Zhao H., Shi J., Qi X., Wang X., Jia J. (2016). Pyramid Scene Parsing Network.

[48] Richards F.J. (1959). A Flexible Growth Function for Empirical Use. J. Exp. Bot..

[49] Hajirad I., Ahmadaali K., Liaghat A. (2025). Crop yield and water productivity modeling using nonlinear growth functions. Sci. Rep..

[50] Hartigan J.A., Wong M.A. (1979). Algorithm AS 136: A k-means clustering algorithm. JSTOR J. R. Stat. Soc..

[51] Punhani A., Faujdar N., Mishra K.K., Subramanian M. (2022). Binning-Based Silhouette Approach to Find the Optimal Cluster Using K-Means. IEEE Access.

